# Army Surgeons in the Field

**Published:** 1861-07

**Authors:** 


ARMY SURGEONS IN THE FIELD.—From the following statement in the *London Lancet* of May twenty-fifth, it will be seen that the position of army surgeons, in actual service, is far from being so safe or pleasant a thing as many suppose:—

"From a recent statement we learn that, from the beginning of the Indian mutiny until the fall of Lucknow, the number of surgeons and assistant-surgeons killed and wounded in action was seventeen. There were killed at Lucknow alone, two; wounded severely, two: total, four. Killed at Jhansi, one; severely wounded, one: both in the same regiment. Of the Arrah party, after the other two officers were killed, Assistant-Surgeon Clarke, of the 35th Regiment, commanded until he was himself mortally wounded. It was Surgeon Hone, (Victoria Cross,) of the 90th Regiment, who commanded the party of doolies when cut off from the column at Lucknow; and it was Assistant-Surgeon Wilson, of the 7th Regiment, who saved the life of the Duke of Cambridge, at Inkermann. It is, then, fairly asked, why are not army surgeons equally rewarded with other officers, since they are equally exposed? Why should there be no brevet, which is at least harmless in its operation? There was one in the late Company's service at last, and we concur in the regret expressed that it is not still in force."

